# Tensile and Ablation Properties of Fiber-Reinforced Si-Modified Phenolic Aerogel Composites

**DOI:** 10.3390/gels12060473

**Published:** 2026-05-29

**Authors:** Junjie Xu, Hui Gao, Jianlong Chang, Lijun Lei, Feng Liu, Pengyong Xie, Yuan Cao

**Affiliations:** 1 School of Mechanical and Electrical Engineering, North University of China, Taiyuan 030051, China; x1239297433@163.com; 2 School of Materials Science and Engineering, North University of China, Taiyuan 030051, China; 18809502933@163.com; 3 School of Energy and Power Engineering, North University of China, Taiyuan 030051, China; leilijun@nuc.edu.cn (L.L.); feng.liu@nuc.edu.cn (F.L.); xiepy230301@nuc.edu.cn (P.X.); 4 Naval Research Academy, Beijing 100161, China; cy_cxyj2022@163.com

**Keywords:** nanopores, phenolic aerogel composites, fiber density, ablation properties, mechanical properties

## Abstract

This study realizes the synergistic improvement in mechanical properties and ablation resistance of Si-modified phenolic aerogel composites with preserved lightweight characteristics and excellent thermal insulation. The resin matrix forms a uniform nanoporous structure, providing prominent thermal insulation performance. The composite with a fiber density of 0.62 g/cm^3^ has a low thermal conductivity of 0.086 W/(m·K). The material exhibits reliable tensile strength within a wide temperature range, and its tensile strength rises significantly with an increase in fiber density. The composite with a fiber density of 0.62 g/cm^3^ delivers a tensile strength of 129 MPa at 20 °C and 102 MPa at 300 °C, which are 79.4% and 122.2% higher than those of the composite with a fiber density of 0.36 g/cm^3^. In addition, methyltriethoxysilane and quartz fiber knitted felts form in situ SiO_2_ and SiC ceramic cladding layers under high-temperature ablation, effectively enhancing the ablation resistance of the composites. Higher fiber density greatly reduces the linear ablation rate. With an oxygen flow of 950 L/h and acetylene flow of 700 L/h, the linear ablation rate of the composite with a fiber density of 0.62 g/cm^3^ is only 0.13 mm/s, 23.1% lower than the composite with a fiber density of 0.36 g/cm^3^.

## 1. Introduction

With the rapid development of the aerospace field, various supersonic and hypersonic vehicles have emerged. This field is not only the core competition area of cutting-edge technologies among countries but also an important carrier demonstrating comprehensive national strength [1,2,3]. At present, aerospace vehicles and supersonic aircraft continue to develop towards higher speed, longer endurance and higher maneuverability, which is accompanied by an increasingly significant aerodynamic heating effect and puts forward strict requirements for the ablation resistance of aircraft [4,5]. Currently, the most widely used thermal protection materials are mainly divided into two categories: ablative materials and non-ablative materials [6,7]. Among them, remarkable advantages are shown by ablative thermal protection materials due to their unique thermal protection mechanism. Under extreme high-temperature environments, materials consume and dissipate a large amount of heat through a series of physical and chemical changes such as melting, sublimation and thermal decomposition [8,9,10]. These materials possess the core advantages of high efficiency, economy and safety, which have become a key thermal protection technical scheme suitable for extreme high-temperature working conditions.

Significant advantages such as low preparation cost, lightweight, high specific modulus and high energy efficiency are exhibited by phenolic resin composites. They are widely used in the lightweight design of aircraft equipment and play an important role in improving the flight speed and maneuverability of aircraft. Lightweight design of aircraft is the core link to improve flight efficiency, optimize energy consumption performance and enhance maneuverability [11]. With the continuous deepening and development of research in the field of thermal protection materials, new thermal protection materials with both lightweight advantages and excellent comprehensive performance are gradually emerging. For example, phenolic aerogel composites prepared by sol–gel and drying technologies have abundant nanoporous structures, which can markedly improve the thermal insulation effect [12,13,14,15]. They are often used as materials with both ablation resistance and efficient thermal insulation under extreme service conditions [16]. Meanwhile, relevant studies have verified that structural heterogeneity can realize the simultaneous strengthening and toughening effect of composite materials, which has established a sound research precedent for exploring the reinforcement mechanism of porous heterogeneous structures [17]. Similar to the energy attenuation mechanism in acoustic metamaterials, where porous heterogeneous structures change wave propagation paths and promote waveform transformation to enhance energy dissipation [18], the nanoporous structure of phenolic aerogels reduces the heat transfer rate by restricting gas-phase thermal conduction and prolonging solid-phase heat transfer paths, thereby achieving efficient thermal insulation. Although phenolic aerogel composites have excellent thermal insulation performance, their internal nanoporous structures bring two prominent problems. The interfacial bonding strength between the resin matrix and fibers is reduced, and the continuity of the resin matrix is weakened. A decline in the overall mechanical properties of the composites directly results from these problems [19,20]. Under high mechanical load conditions, the main structure of the aircraft is prone to large deformation, and the relevant load will be directly transmitted to the thermal protection layer. Limited by insufficient mechanical properties, materials are unable to bear such loads and are prone to cracks. Once cracks form, they become channels for high-temperature gas to invade the interior of the material, resulting in a significant attenuation of ablation resistance and thermal insulation effect of the material, and then pose a serious threat to the overall structural safety of the aircraft. Therefore, on the premise of ensuring the excellent thermal insulation performance of composites, synchronously improving their mechanical properties and ablation resistance has become an urgent key scientific and engineering problem to be solved.

Microporous structure design has been verified to simultaneously tailor the stiffness and energy dissipation characteristics of materials [21], which provides a new perspective for addressing the inherent trade-off between thermal insulation and mechanical strength in phenolic aerogel composites. Notably, a dual-strategy approach combining reinforcement-phase incorporation and induced porosity has been demonstrated to effectively regulate porous structure and mechanical performance synchronously [22], which indicates that tailoring reinforcing fibers provides an effective way to adjust the comprehensive performance of composites. The interfacial bonding characteristics between fibers and the resin matrix can be effectively improved by increasing fiber volume fraction [23,24,25], changing fiber types [20] and modifying fiber surfaces [26,27,28], so as to affect the properties of composites [29,30]. Fiber–resin interfacial bonding and internal stress transfer within materials are governed by fiber preform density. Accordingly, rational regulation of fiber preform density is expected to mitigate the deficiencies in resin matrix continuity and interfacial bonding strength, thereby synergistically enhancing the mechanical and anti-ablation properties of composites. At present, research on the modification and performance optimization of reinforcing fibers is relatively systematic, but systematic research on the influence of fiber preform density on the properties of phenolic aerogel composites is still lacking. The continuity of the resin matrix and the stress transfer path are changed by the nanoporous structure, and the effect of fiber preform density on traditional phenolic resin composites is difficult to apply to phenolic aerogel composites. Therefore, research on the fiber density of phenolic aerogel composites is of great significance for application in the field of aerospace thermal protection materials.

The thermal insulation capacity of phenolic aerogel composites can be effectively improved by nanoporous structures while maintaining lightweight advantages, which satisfies the extreme thermal protection demands of aircraft. Nevertheless, nanopores weaken interfacial bonding and disrupt the continuity of the resin matrix, leading to the degradation of mechanical and ablation resistance, making it difficult to meet the comprehensive service requirements of thermal insulation, load-bearing and anti-ablation performance. In this paper, phenolic aerogel composites with three fiber densities are prepared by the resin transfer molding process (RTM) [31,32]. The fiber density of needle-punched preforms is changed by adjusting the ply thickness of fiber fabric and mesh tires [33,34] and adjusting the needle-punching density. Then, the microstructure, mechanical properties, thermal resistance and ablation properties of the three composites are systematically tested, aiming to clarify the influence law of fiber preform density on the comprehensive properties of phenolic aerogel composites. Beyond aerospace thermal protection systems, the multifunctional characteristics of the as-prepared composites enable broader application prospects. The combined advantages of controllable porous structure, favorable mechanical robustness, and high-temperature stability render the material promising for impact-resistant protective components, lightweight load-bearing thermal insulation structures, and load-bearing components under extreme thermal–mechanical coupling environments.

## 2. Results and Discussion

### 2.1. Basic Physical Properties of Composites

**Figure 1:**
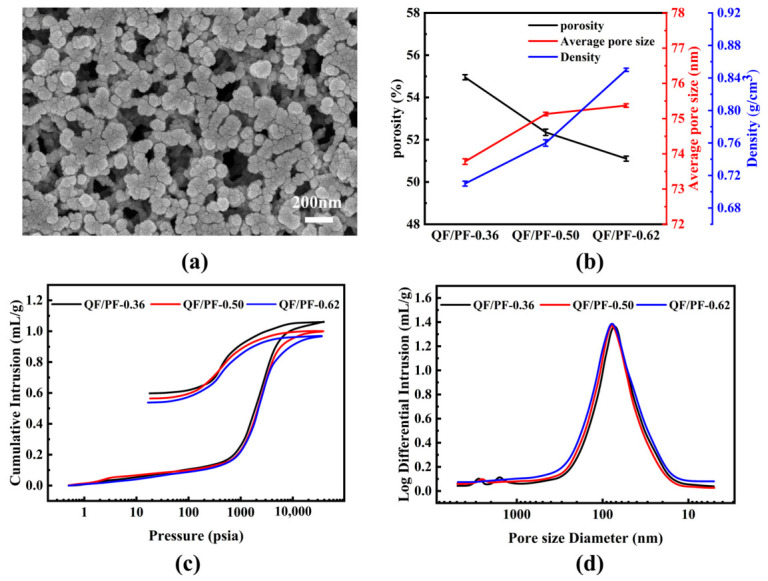
Micromorphology of resin matrix microspheres and pore characteristics of phenolic aerogel composites with three fiber densities. (**a**) Nanoresin microspheres. (**b**) Density and pore size of composites. (**c**) Mercury intrusion and extrusion curves. (**d**) Pore size distribution curve.

The micromorphology and pore distribution of the phenolic aerogel composites with three fiber densities are displayed in Figure 1. The resin matrices of the three composites exhibit similar morphologies, consisting of nanoscale microspheres stacked into a continuous three-dimensional network. As shown in Figure 1a, the micromorphologies of the resin microspheres are comparable across the three samples, indicating that variations in the fiber density of the preform barely affect the morphology of the resin microspheres.

The mercury intrusion test reveals that the change in fiber density affects the porosity of the composites but has no significant effect on the average pore size. A highly consistent trend is exhibited by the mercury intrusion and extrusion curves of the three composites. When the pressure is below 1000 Psia, the curve increases slowly, which proves that the content of micron-sized pores in the material is low. When the pressure is above 1000 Psia, the mercury intrusion curve rises sharply, and mercury is pressed into the pores in large quantities. Since a steep, concentrated intrusion stage only appears when a large number of pores with similar diameters are penetrated at the same time, this rapid rising trend directly proves that these pores are uniform in size and located at the nanoscale. In addition, the average pore size curves of the three composites overlap highly, and the average pore size is in the range of 73–76 nm, which indicates that the change in fiber density has no obvious effect on the pore structure of composites, and its pore structure is mainly dominated by the properties of phenolic resin itself. The porosities of QF/PF-0.36, QF/PF-0.50 and QF/PF-0.62 are 55.1%, 52.4% and 51.1%, respectively. The porosity of the composites is affected by fiber density because the change in fiber density affects the volume proportion of the resin matrix in the composites. The higher the fiber density, the larger the space occupied by fibers, and the correspondingly smaller space occupied by the resin matrix. Since pores are mainly formed and exist in the resin matrix, the rise in fiber density leads to a reduction in porosity. The densities of QF/PF-0.36, QF/PF-0.50 and QF/PF-0.62 are 0.71 g/cm^3^, 0.76 g/cm^3^ and 0.85 g/cm^3^, respectively. The density of the composites is 19.7% higher in QF/PF-0.62 than in QF/PF-0.36 with increasing fiber needle-punched felt density, but the densities of the three materials are all less than 1 g/cm^3^, which is still lower than most traditional phenolic resin composites and has the advantage of being lightweight. At the same time, the growth in density often brings better mechanical properties [35], which can better adapt to high-load service environments.

### 2.2. Thermal Properties of Composites

**Figure 2:**
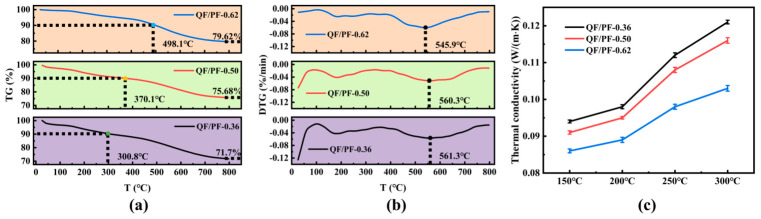
Thermal stability and thermal conductivity of phenolic aerogel composites with three fiber densities. (**a**) TG curves. (**b**) DTG curves. (**c**) Thermal conductivity.

The thermogravimetric and thermal conductivity test results of the phenolic aerogel composites with three different fiber densities are presented in Figure 2. The residual carbon rates of QF/PF-0.36, QF/PF-0.50 and QF/PF-0.62 at 800 °C are 71.1%, 75.68% and 79.62%, respectively. The residual carbon rate increases gradually with the increase in fiber density, with an increment of 8.52% from QF/PF-0.36 to QF/PF-0.62, which effectively improves the structural shape retention and ablation resistance of the materials.

Multi-stage mass loss characteristics are exhibited by the composites during heating. The stage of 0–200 °C is dominated by the volatilization of residual solvents and the escape of incompletely crosslinked oligomers. In the range of 200–600 °C, the resin matrix undergoes thermal decomposition, the crosslinking bonds of phenolic resin molecular chains break, and small molecular gases such as CH_4_, CO_2_ and H_2_O are released. In the stage of 600–800 °C, the residual resin undergoes further dehydration and condensation reactions, and the thermally decomposed resin matrix gradually transforms into a dense carbonaceous skeleton. This can be attributed to the fact that the higher the fiber proportion, the less resin matrix available for thermal decomposition reactions in composites. Quartz fibers with excellent thermal stability are kept almost unchanged within the test temperature range, by which the overall mass loss of the composites is effectively inhibited, and the composites with higher fiber density are finally endowed with more excellent thermal stability. The thermal weight loss characteristic temperatures of the three composites are presented in Table 1. The temperatures corresponding to 5% and 10% mass loss of QF/PF-0.62 are 343.8 °C and 498.1 °C, respectively, which are much higher than those of QF/PF-0.36 and QF/PF-0.50. The temperature corresponding to 5% mass loss is 103.1% higher than that of QF/PF-0.36, while the temperature associated with 10% mass loss exceeds that of QF/PF-0.36 by 65.6%. In addition, the maximum thermal decomposition temperatures of the three composites are concentrated in the range of 545–562 °C with relatively small differences. Nevertheless, they still follow the rule that a higher fiber density corresponds to a lower maximum decomposition temperature, which further confirms that increasing fiber density exerts an inhibitory effect on the mass loss of composites.

The thermal conductivity of the composites with three fiber densities at different temperatures is compared in Figure 2c. The results show that the thermal conductivity of the three composites increases with the increase in test temperature. Across the whole temperature range, QF/PF-0.36 has the highest thermal conductivity, followed by QF/PF-0.50, and QF/PF-0.62 has the lowest, showing a rule that the higher the fiber density, the lower the thermal conductivity. At the same time, it is found that the thermal conductivities of QF/PF-0.36 and QF/PF-0.50 are close at four temperatures, while the thermal conductivity of QF/PF-0.62 is significantly lower than those of the other two materials, indicating better thermal insulation ability. When the test temperature rises from 200 °C to 250 °C, the thermal conductivity of the three composites increases markedly, while the rise in the thermal conductivity of the three materials diminishes significantly when the test temperature rises from 150 °C to 200 °C. Taking QF/PF-0.36 as a typical example, its thermal conductivity is 0.094 W/(m·K) at 150 °C and slightly increases to 0.098 W/(m·K) at 200 °C, with an increase of only 4.26%. In sharp contrast, the thermal conductivity jumps to 0.112 W/(m·K) at 250 °C, representing a remarkable increase of 14.29% compared with that at 200 °C, which clearly verifies the significantly accelerated growth trend of thermal conductivity above 200 °C. This can be ascribed to solvent volatilization and oligomer escape in the composites at 150 °C and 200 °C. The resin matrix has not yet started thermal decomposition or is ready for thermal decomposition, and the resin matrix and fiber structure in the material are basically stable. Although the volatilization process of solvents and oligomers is more intense at 200 °C, the gas molecular weight is small, the flow inside the material is weak, and the contribution to convective heat transfer is limited, so the thermal conductivity increases gently. Based on the theory of granular–fluid interactions in dense complex fluids proposed by Sun et al., the migration and heat transfer behavior of internal gas are closely related to the structural continuity of the system [36]. At 200 °C, the resin matrix maintains good structural integrity, which provides strong interfacial restriction and high flow resistance, thus inhibiting the convective transport of pyrolysis gas. When the temperature rises to 250 °C, massive thermal decomposition of the resin occurs, producing a large amount of pyrolysis gas inside the material. Heat transfer to the interior of the material is markedly accelerated by these gases through convective heat exchange. At the same time, the resin matrix decomposes and is damaged under the action of heat, and the integrity of the internal structure of the material decreases, which reduces the interfacial friction and flow resistance significantly. Such structural degradation will markedly enhance the two-phase convective motion inside the material, thereby causing a sharp rise in thermal conductivity and making it easier for hot air flow to invade the interior of the material. The two aspects work together and finally lead to a sharp rise in the thermal conductivity of composites. The thermal conductivity values of composites with three fiber densities at different temperatures are recorded in Table 2, and the improvement range of thermal conductivity of QF/PF-0.62 compared with QF/PF-0.36 at different temperatures is listed in Table 3. With the increase in temperature, the improvement range of thermal conductivity of QF/PF-0.62 compared with QF/PF-0.36 gradually expands. Particularly, when the temperature rises from 200 °C to 250 °C, the growth of thermal conductivity increases significantly from 0.009% to 0.014%, which may be related to the large difference in resin thermal decomposition degree at the two temperatures.

### 2.3. Tensile Properties of Composites

**Figure 3:**
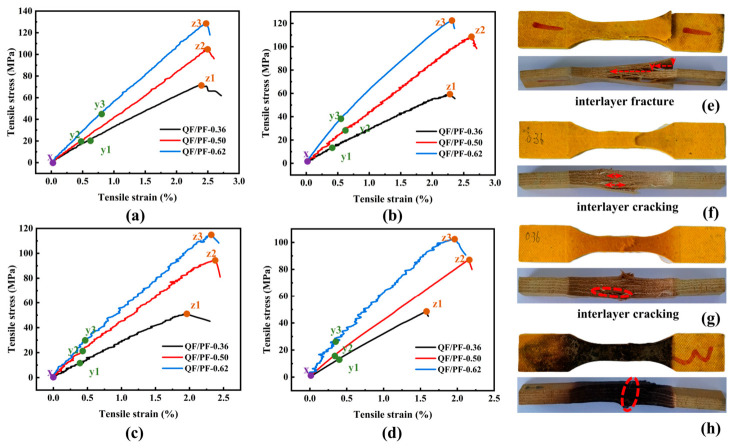
Tensile stress–strain curves and tensile fracture morphologies of phenolic aerogel composites with three fiber densities. (**a**–**d**) Tensile stress–strain curves at 20 °C, 100 °C, 200 °C, and 300 °C. (**e**–**h**) Tensile fracture morphologies of QF/PF-0.36 at 20 °C, 100 °C, 200 °C, and 300 °C.

The tensile stress–strain curves of the composites with three different fiber densities and the tensile fracture morphology of QF/PF-0.36 at different temperatures are presented in Figure 3. A similar three-stage variation rule is observed in the stress–strain curves of the three composites at different temperatures. The stress undergoes an elastic stage, a plastic stage, and finally a failure stage with the increase in tensile load. The x-y stage is the elastic stage, in which the stress grows linearly with the increase in strain. Due to the low tensile load, it is not enough to damage internal defects, such as cracks and pores of the composites, and the interfacial bonding between fibers and the resin matrix [12,37]. The original shape of the composites can be restored through elastic deformation after unloading. The results illustrate that QF/PF-0.62 has the largest slope of the stress–strain curve, followed by QF/PF-0.50 and QF/PF-0.36. The larger the slope of the curve, the stronger the ability of the material to resist deformation. Therefore, the overall mechanical bearing performance of QF/PF-0.62 is better than that of the other two composites at this stage. With the continuous growth in tensile load, the material enters the y-z plastic stage. Internal defects of composites increase continuously along the tensile direction, cracks expand continuously, pores undergo irreversible deformation, and the interface between fibers and the resin matrix debonds gradually, which slows down the growth rate of stress with strain. Further analysis shows that with the increase in strain, QF/PF-0.62 still has the fastest stress rise rate, indicating that its tensile strength and deformation resistance are still the best at this stage. When the load continues to rise, a large number of internal defects in the composites initiate and connect; cracks expand and penetrate continuously, finally leading to macroscopic fracture, and the stress drops sharply. Based on the cohesive zone model (CZM) and interfacial fatigue behavior theory, progressive interfacial debonding and crack propagation are the mechanisms responsible for the rapid decline in bearing capacity and macroscopic brittle fracture [38].

A similar change trend is exhibited by the fracture mode of the phenolic aerogel composites with three fiber densities as the temperature grows. QF/PF-0.36 is used as an example in this paper to illustrate the change rule of the fracture behavior of these three composites with temperature. The morphology of QF/PF-0.36 after tensile fracture at 20 °C, 100 °C, 200 °C and 300 °C is presented in Figure 3e–h, respectively. It can be seen that with the elevation in temperature, the interlaminar fracture between the fiber fabric and mesh tires gradually weakens. At 20 °C, interlaminar debonding is obvious, and the debonding area exceeds one-third of the whole sample. With the increase in temperature, interlaminar debonding gradually weakens, and the debonding area decreases markedly. This can be attributed to the accelerated thermal decomposition rate of the resin with increasing temperature, which weakens the connection between resin microspheres and between resin microspheres and fibers. Under load, fibers and the resin matrix are easier to break, so most fractures occur in the surface layer direction, the tension transmitted to the interlayer is reduced, and the occurrence of interlaminar fracture is thus alleviated.

**Figure 4:**
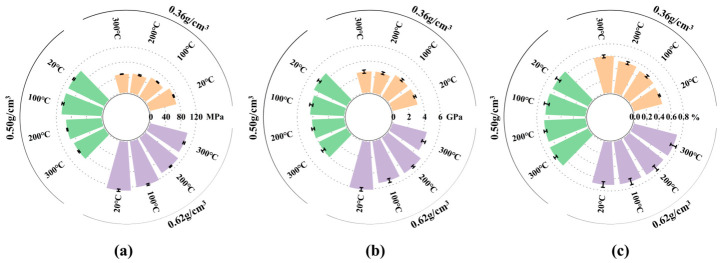
Tensile properties of phenolic aerogel composites with three fiber densities. (**a**) Tensile strength. (**b**) Tensile modulus. (**c**) Elongation at break.

The tensile strength, tensile modulus and elongation at break of the phenolic aerogel composites with three different fiber densities are presented in Figure 4. At different temperatures, the tensile strength of the composites increases with the increase in fiber density. The growth of fiber density is realized by increasing the layers of fiber fabric and mesh tires in the preform. When the preform bears the tensile load in the plane of the fiber fabric and mesh tires, the tensile strength of the fiber preform can be effectively improved by increasing the surface layers. For this reason, the preform with excellent tensile properties retains its properties after being made into composites, finally leading to the improvement in the tensile properties of the composites. In addition, the rise in fiber density means that the spatial proportion of fibers increases after being made into composites. Since the tensile properties of fibers are significantly better than those of the resin matrix, this higher fiber proportion can directly endow composites with more excellent tensile properties. Therefore, excellent tensile properties are exhibited by composites with high fiber density, enabling better adaptation to high-load service environments.

The tensile strength of the phenolic aerogel composites with three fiber densities at different temperatures is listed in Table 4. Table 5 demonstrates the improvement range of tensile strength of QF/PF-0.62 compared with QF/PF-0.36 at each temperature. The attenuation of tensile strength of the three composites when the temperature rises from 20 °C to 300 °C is displayed in Table 6. When the temperature rises from 20 °C to 300 °C, the improvement ranges of tensile strength of QF/PF-0.62 compared with QF/PF-0.36 are 79.4%, 107.1%, 116.5% and 122.2%, respectively, showing a trend of gradual expansion with the increase in temperature. During heating, the composites experience volatilization of residual solvents and escape of low-molecular-weight linear polymers, and the resin matrix undergoes a thermal decomposition reaction. These processes continuously generate and release large amounts of small-molecule gases inside the composites. Since these gases cannot escape rapidly in a short time, they accumulate locally and lead to a significant increase in internal pressure. The increased internal pressure further promotes the expansion and propagation of the original defects inside the material, which eventually causes the degradation of the tensile properties of the composites. According to the micromechanical mechanism of thermoplastic composites under thermal cycling and cyclic loading, temperature elevation induces obvious irreversible deformation and cumulative microdamage within the resin matrix [39]. Such thermal-induced irreversible evolution accelerates defect propagation and weakens the fiber–matrix interface, which is consistent with the tensile performance degradation of composites at elevated temperatures observed in this study. The more significant improvement in QF/PF-0.62 at higher temperatures just indicates that the increase in fiber density can greatly reduce the tensile performance decline caused by the increase in high-temperature-induced defects.

### 2.4. Ablation Properties of Composites

**Figure 5:**
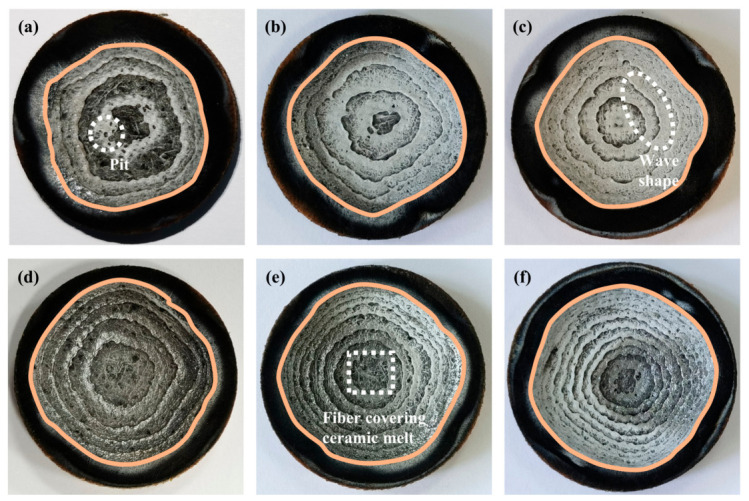
Ablation morphologies of phenolic aerogel composites with three fiber densities. (**a**–**c**) Ablation morphologies under case 1. (**d**–**f**) Ablation morphologies under case 4.

To compare the ablation performance of the composite materials under different oxygen and acetylene flow rates, four sets of flow parameters for oxygen and acetylene are selected for testing, as listed in Table 7. The oxyacetylene ablation morphologies of the three composites are presented in Figure 5. It can be found that under both case 1 and case 4 conditions, with the increase in fiber density, the number of exposed fibers and ablation holes in the ablation crater decreases significantly, and the coverage of the ceramic coating layer in the ablation crater rises obviously. Under the action of ablation scouring, each layer of fiber fabric in the composite is ablated, forming a wave-shaped ablation edge in the ablation crater. With the increase in fiber density, the number of layers of fiber fabric and mesh tires grows accordingly, the compactness of the fiber preform is considerably improved, and the anisotropy of the fiber fabric in the surface layer direction gradually weakens, which makes the morphology of the ablation edge more regular. The uneven ablation rate caused by excessive anisotropy can be effectively improved by this structural feature. An uneven ablation rate will lead to excessive local ablation, which will further accelerate the ablation rate in the in-plane and interlaminar directions of the area and directly promote the rise in the overall ablation rate of the material.

At the same time, it can be seen from Figure 5a that local excessive ablation accelerates the recession of the ablation edge, reduces the coverage area of the ceramic coating layer, and further increases the risk of exposure of the resin matrix and fibers. This makes it easier for hot air flow to invade the interior of the material, accelerates the thermal decomposition of the resin matrix and the melting of fibers, and finally leads to a significant increase in the ablation rate of the composites. It is indicated by the above analysis that the uniformity of the material can be enhanced by increasing fiber density, the uneven ablation rate during ablation can be markedly improved, and the ablation resistance of the material can be improved accordingly.

**Figure 6:**
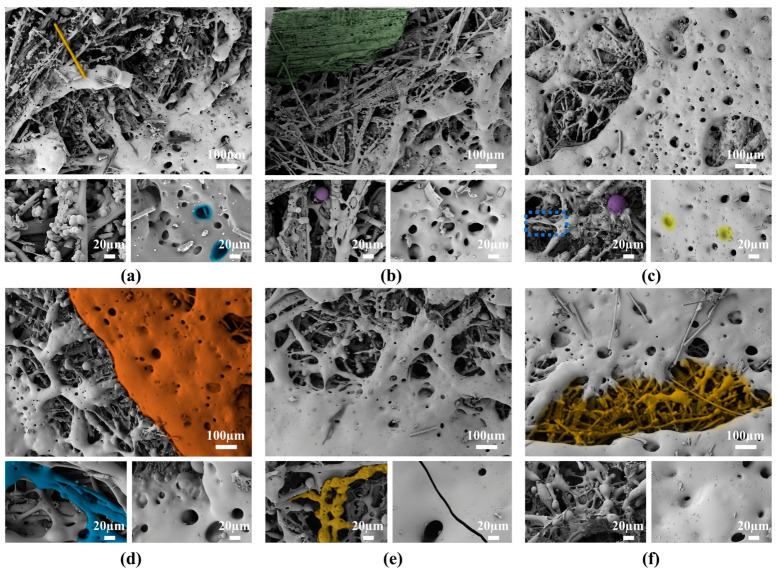
Ablation micromorphologies of phenolic aerogel composites with three fiber densities. (**a**–**c**) Ablation micromorphologies under case 1. (**d**–**f**) Ablation micromorphologies under case 4.

The ablation micromorphology of the phenolic aerogel composites with three fiber densities is presented in Figure 6. In each figure, the upper part is the overall ablation morphology, the lower left corner is the fiber morphology after ablation, and the lower right corner is the ceramic coating morphology after ablation. From the overall morphology, it can be seen that there are ceramic coating layers and exposed quartz fibers on the ablation surfaces of the three composites. However, with the increase in fiber density, the coverage area and thickness of the ceramic coating layers increase markedly, which can effectively block the penetration of high-temperature gas into the material, thus improving the ablation resistance of the material. As observed in typical high-temperature protective coatings, the ceramic layer generated in this study also follows a universal thermal protection mechanism [40]. During ablation, the formed ceramic phases construct a dense protective barrier, which effectively isolates oxygen penetration and resists the scouring of high-speed airflow. Quartz fibers exposed to high-temperature and high-speed air flow are seriously eroded, melted and fractured, resulting in the loss of their original skeleton support function. The reinforcement effect of fibers on the resin matrix and carbon layer is weakened by the damage to the fibers. Under the shear force of air flow, the carbon layer without fiber support is easily scoured and peeled off, leading to a decline in the overall ablation resistance of the material. By comparing the ablation morphologies of the three composites, it can be found that there are fewer exposed fibers in the samples with higher fiber density. This phenomenon results from the greater number of melting sources provided by high fiber density. At high temperatures, a large number of quartz fibers undergo a melting phase transition, and the generated ceramic melt spreads into a film on the ablation surface. This process not only removes a lot of heat through phase change heat absorption but also further hinders the invasion of hot air flow. In addition, part of the melt can penetrate into the pores of the carbon layer, playing a role in connecting fibers and the carbon layer and enhancing the ability of the structure to resist aerodynamic shear. Such microstructural evolution and protective behavior are highly consistent with the typical regulation mechanism of conventional ceramic coating systems during ablation, confirming the universal applicability of this thermal protection mechanism [41]. It can be seen that increasing fiber density can improve the ablation resistance of the material through the heat absorption effect of fiber melting phase transition and the blocking effect of hot air flow. By comparing the fiber morphologies of case 1 and case 4, it can be seen that with the increase in oxyacetylene flow, the ablation and melting of the fibers become more and more significant. The melted fibers lose their original morphology and show a wire-drawing-like morphology, as exhibited in Figure 6d–f, after heating and cooling. Although heat transfer can be effectively alleviated by the phase change heat absorption process of fiber melting, the invasion of hot air flow into the composite can be accelerated by serious damage to the fiber structure, resulting in the decline in its ablation resistance.

**Figure 7:**
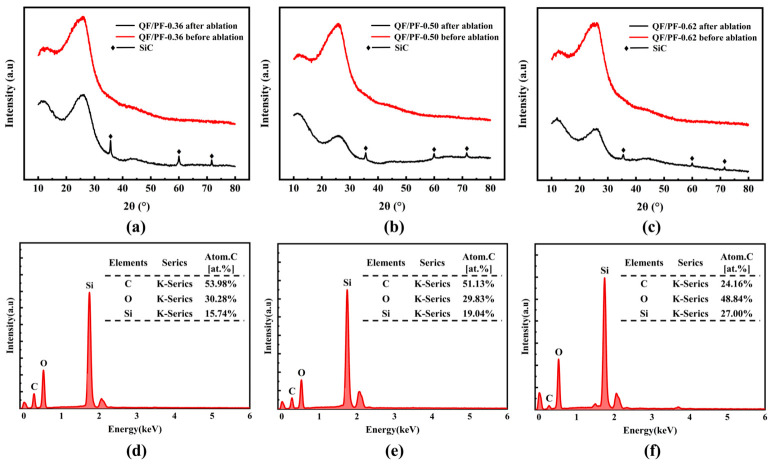
Element analysis of the ablated surfaces of phenolic aerogel composites with three fiber densities. (**a**–**c**) XRD of QF/PF-0.36, QF/PF-0.50, and QF/PF-0.62, respectively. (**d**–**f**) EDS of QF/PF-0.36, QF/PF-0.50, and QF/PF-0.62, respectively.

XRD and EDS analyses are carried out on the ablation surface to analyze the crystalline phase structure and element distribution, as shown in Figure 7. The three composites use the same phenolic resin as the matrix and the same type of fiber as the reinforcement. Only the density of quartz fibers is different, so the crystalline phase structure formed after ablation is highly similar. From the XRD pattern, it can be seen that the diffraction peak shape of all samples changes consistently before and after ablation. Before ablation, there are no obvious diffraction peaks in the XRD patterns of all samples. After ablation, characteristic diffraction peaks appear at 2θ = 36°, 60° and 72°, which correspond to the (1 1 1), (2 2 0) and (3 1 1) crystal plane diffractions of SiC, respectively [30]. At the same time, a broadened diffuse peak appears near 2θ ≈ 25°, which is a typical characteristic diffraction peak of amorphous SiO_2_ [42]. The above results confirm that SiO_2_ and SiC are the main ceramic phases formed on the ablation surface of the composites.

The formation path of the ceramic phase is further analyzed. The source of SiO_2_ mainly includes two parts. One is the melting phase transition of quartz fibers in the high-temperature environment of oxyacetylene ablation, in which the amorphous structure is retained after cooling. The other is the oxidation reaction of Si-modified groups, in which silicon is finally converted into amorphous SiO_2_. The formation of SiC is closely related to the special environment during ablation. In the early stage of ablation, the phenolic resin matrix undergoes intense thermal decomposition at high temperatures, generating a large amount of pyrolysis gas. When these gases escape from the composite surface, they form a dense gas-phase barrier at the interface, which not only effectively blocks the invasion of external high-temperature gas into the material but also isolates oxygen and builds a local high-temperature anaerobic environment on the material surface. In this environment, the amorphous SiO_2_ prepared in the early stage undergoes a reduction reaction with activated carbon produced by resin pyrolysis and finally generates SiC. The specific reaction is presented in Equation (1). However, when SiC is exposed to an oxygen atmosphere, SiC undergoes an oxidation reaction to produce SiO_2_ and CO_2_, as shown in Equation (2).

SiO_2_ (s) + 3C (s) → SiC (s) + 2CO (g) (1)

SiC (s) + 2O_2_ (g) → SiO_2_ (s) + 2CO_2_ (g) (2)

EDS is used to analyze the element composition and distribution of the ablation surfaces of the three composites. All three composites contain three elements: C, O and Si. However, the atomic percentages of C, O and Si in the different samples are different, which are represented by C_C0_, C_O_ and C_Si_, respectively, as illustrated in Figure 7d–f. Specifically, with the growth in fiber density, the Si element content on the ablation surface increases significantly: compared with QF/PF-0.36, the Si content is increased by 3.30% for QF/PF-0.50 and by 11.26% for QF/PF-0.62. Since the generated ceramic coating layer is SiO_2_ and SiC, the increase in the Si element content directly reflects the increase in the total amount of the ceramic phase on the ablation surface. The more ceramic coating layers, the more obvious the effect of resisting hot air erosion. This result is consistent with the phenomenon that the coverage area of the ceramic coating layer grows with the growth in fiber density observed in Figure 5. In a high-temperature oxidation environment, the O element mainly combines with the Si element to form SiO_2_, so it can be considered that all O elements participate in the formation of SiO_2_. Accordingly, the formation amount of SiO_2_, denoted as C_SiO_2__, can be calculated by the O element content, and the specific expression is shown in Formula 3. The remaining Si element reacts with C to generate SiC, and its content, denoted as C_SiC_, is displayed in Formula 4. Finally, the unreacted residual C element is the exposed carbon layer tissue on the ablation surface, and its content, denoted as C_C1_, is calculated by Formula 5.

Based on the above calculation method, the atomic percentages of SiO_2_, SiC and C in the carbonized layer on the ablation surfaces of the three composites are obtained, as shown in Table 8. It can be noticed that the SiO_2_ content on the ablation surface of QF/PF-0.62 is 61.29% and 63.73% higher than that of QF/PF-0.36 and QF/PF-0.50, respectively. At the same time, the total amount of SiC and SiO_2_ on the ablation surface of QF/PF-0.50 is also considerably higher than that of QF/PF-0.36. Observing the atomic percentage of C in the carbonized layer, it is found that with the increase in fiber content, the exposed carbon layer tissue decreases significantly. Because the exposed carbon layer structure is loose and easily forms open air flow channels, hot air flow can easily pass through the carbon layer and invade the interior of the material, thus weakening the ablation resistance of the material. For advanced high-temperature structural materials, the introduction of metallic phases and dendritic reinforcements has been validated as a universal design strategy to enhance thermal stability and deformation resistance under extreme service conditions [43]. This strengthening mechanism is analogous to the role of reinforcing fibers in phenolic aerogel composites: increasing fiber density helps improve the compactness and coverage of the SiO_2_/SiC ceramic layer, reduce structural anisotropy, and suppress ablation non-uniformity. Therefore, increasing fiber density significantly improves the ablation resistance of the composites.

C_SiO_2__ = C_O_/2 (3)

C_SiC_ = C_Si_ − C_SiO_2__ (4)

C_C1_ = C_CO_ − C_SiC_ (5)

**Figure 8:**
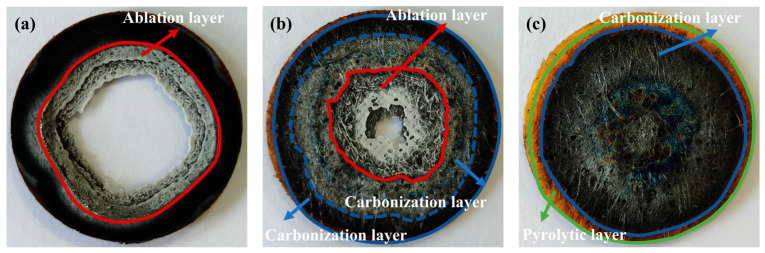
Ablation morphologies of QF/PF-0.36 sample under case 1. (**a**) Ablation surface. (**b**) Morphology of 2 mm from the top. (**c**) Morphology of 3 mm from the top.

In order to analyze the existence state of ceramic melt in the ablation layer, carbonized layer and pyrolysis layer, as well as the difference in the ablation morphology of each layer, the QF/PF-0.36 sample after ablation under the case 1 condition is sliced. The ablation morphology of the ablation surface, the slice 2 mm from the sample top, and the slice 3 mm from the sample top are illustrated in Figure 8a–c, respectively. The study finds that ceramic melt is mainly distributed in the ablation layer and carbonized layer. The ceramic melt in the ablation layer spreads into a film on the surface of the carbon layer, completely covering the carbon layer that should have been exposed, which can effectively weaken the erosion of hot air flow on the interior of the material. The ablation layer in Figure 8b is the missing middle part of Figure 8a. Even though there are exposed fiber structures in this area, the fiber surfaces are basically wrapped by ceramic melt, so the fibers can maintain their original reinforcement effect. The retained skeleton support function of fibers can improve the ability of the material to resist aerodynamic shear, greatly reduce the massive peeling of the carbon layer caused by aerodynamic shear, and thus strengthen the overall ablation resistance of the material.

In Figure 8b, the blue dashed line divides the carbonized layer into two types of regions. The core difference comes from the different ablation temperatures. The region directly affected by oxyacetylene flame has a high ablation temperature, fibers melt to form a silver-white ceramic phase, and the whole region is silver-white. The region with a low temperature has unmelted fibers and is black. In contrast, there is a small amount of ceramic phase on the surface of the carbonized layer in Figure 8c, and the fiber structure is basically intact. It can be inferred that with the increase in sample depth, the ablation effect gradually weakens, the fiber melting phenomenon is significantly reduced, and most fibers can still maintain a complete skeleton structure.

The ablation layer is almost completely covered by the ceramic coating layer; the number of through-holes on the surface is small, and most of them are concentrated in the central area of the ablation crater. The surface of the carbonized layer is distributed with a large number of holes of different sizes. The formation of holes may be attributed to the combined effect of oxyacetylene flame ablation and the escape of internal pyrolysis gas of the material to the surface. Under the scouring of high-temperature and high-speed air flow, the carbonized layer is loose and has low structural strength, so it easily forms holes. At the same time, the internal resin matrix thermally decomposes, generating a large amount of gas, and the carbonized layer structure is further damaged during the escape process, with a large number of holes finally formed. These holes provide penetration channels for external high-temperature air flow, aggravate the pyrolysis reaction of the inner resin, and greatly impair the ablation resistance. Comparing Figure 8b with Figure 8c, it can be seen that the number of holes gradually decreases with the increase in sample depth. This is mainly because the attenuation of heat transfer diminishes the pyrolysis reaction rate; the deep layer is not affected by air flow scouring, and the structural damage is reduced, so the number of holes generated is small. Figure 8c shows that the pyrolysis layer is darker in color than the original layer due to heating, and the bonding strength between fibers and the resin matrix is reduced by resin pyrolysis, causing more fibers to be exposed on the surface of the pyrolysis layer in the form of burrs.

**Figure 9:**
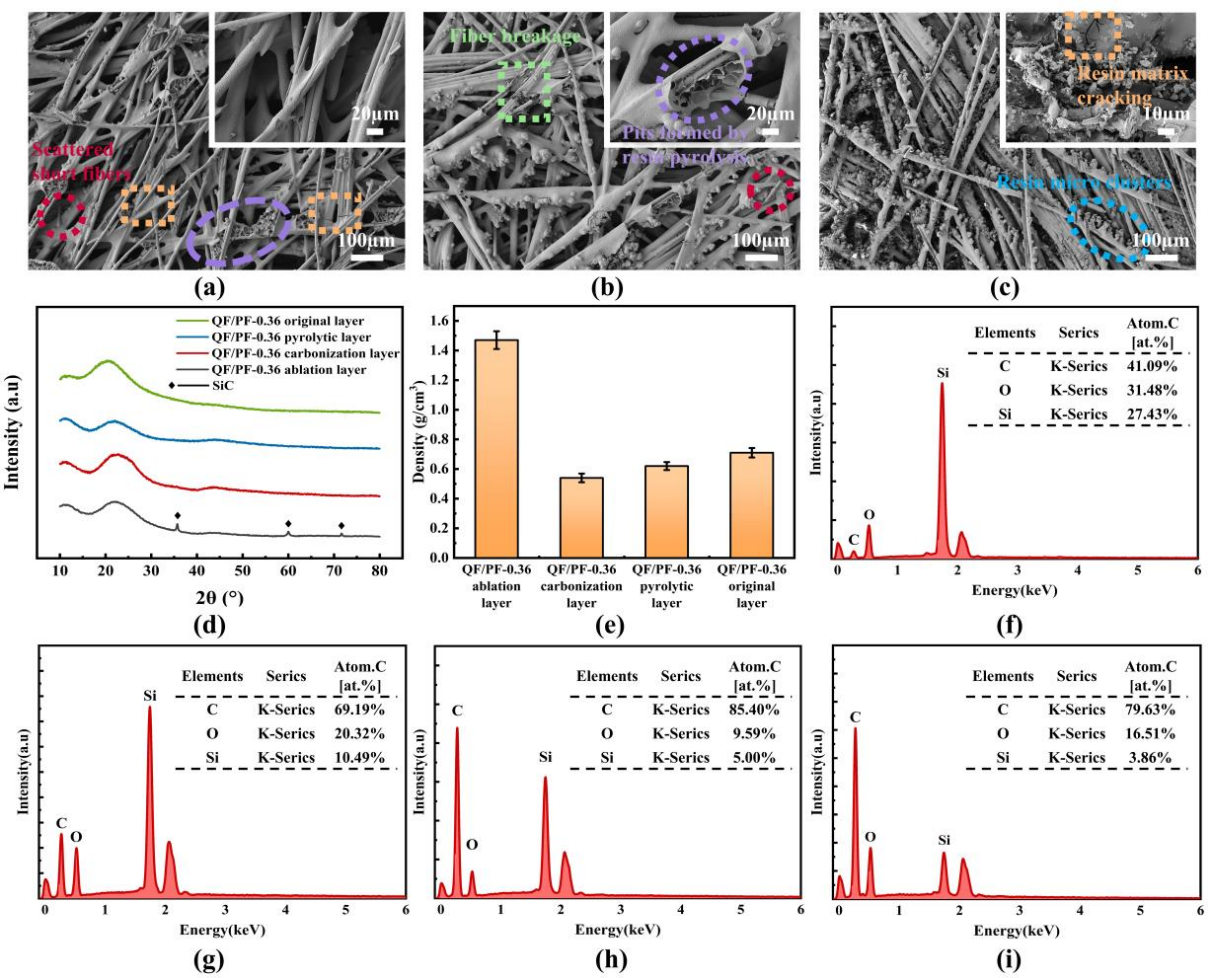
Micromorphologies, XRD analysis, density and EDS analysis of the ablated cross-section of QF/PF-0.36 sample under case 1. (**a**–**c**) Micromorphologies of the ablation layer, carbonized layer and pyrolysis layer, respectively. (**d**) XRD analysis of the ablation layer, carbonized layer, pyrolysis layer and original layer. (**e**) Densities of the ablation layer, carbonized layer, pyrolysis layer and original layer. (**f**–**i**) EDS analysis of the ablation layer, carbonized layer, pyrolysis layer and original layer, respectively.

The micromorphology and element composition of the ablation layer, carbonized layer and pyrolysis layer are analyzed in detail in Figure 9. The ablation layer and carbonized layer demonstrate similar microstructure characteristics. Quartz fibers melt to varying degrees, and a large number of fiber surfaces are covered by ceramic coating layers, especially at fiber intersections, where continuous ceramic films are more likely to form. In addition, scattered short fibers and dense pit structures left by resin matrix pyrolysis on the fiber surfaces can be observed. In addition to the above common characteristics, the ablation layer is directly exposed to a high-temperature flame, so obvious fiber fracture occurs. The ceramic coating layer covering and bridging the fibers is considerably thicker and has a larger coverage area. In contrast, no fiber melting occurs in the pyrolysis layer. Incompletely pyrolyzed resin particles attached to the fiber surface can be clearly observed, and most fibers are still wrapped by the residual resin matrix. XRD analysis is conducted on each layer in Figure 9d. The study finds that the characteristic diffraction peak of SiC only appears in the ablation layer but not in the carbonized layer. Such a phenomenon arises from the loose carbon structure in the carbonized layer, which provides channels for oxygen intrusion, forming an oxygen-rich high-temperature environment within the carbonized layer and preventing the Si element from undergoing a reduction reaction with activated carbon to form SiC.

The density test data of the ablation layer, carbonized layer, pyrolysis layer and original layer are shown in Figure 9e. There are obvious differences in the density of different regions. The ablation layer has the highest density, reaching 1.47 g/cm^3^, which is about twice as high as that of the original layer (0.71 g/cm^3^). The carbonized layer has the lowest density, which is 0.54 g/cm^3^. The density of the pyrolysis layer is 0.62 g/cm^3^, which is between the original layer and the carbonized layer. Since the ablation layer is directly subjected to high-temperature erosion, a continuous and dense ceramic coating layer is generated under the extreme thermal environment. And because the density of the ceramic is much higher than that of the composite, the ablation layer has high-density characteristics as a whole. For the carbonized layer, the resin escapes in gaseous form after pyrolysis, resulting in the transformation of the originally dense phenolic resin structure into a loose carbon structure, and the density is markedly reduced. Although the pyrolysis layer shows no fiber melting phenomenon, part of the resin undergoes a pyrolysis reaction, causing a small amount of mass loss, so its density is between the original layer and the carbonized layer. The EDS analyses of the ablation layer, carbonized layer, pyrolysis layer and original layer are presented in Figure 9f–i, respectively. It can be found that the ablation layer has the highest Si element content, reaching 27.43%. Compared with the Si element content in the carbonized layer, it is increased by 161.49%. This phenomenon is due to the fact that the Si element in the ablation layer spreads into a film in the form of the ceramic phase, covering a large area of the carbon layer tissue, so a large amount of the Si element is observed in the ablation layer. The Si element in the carbonized layer mainly comes from exposed fibers and ceramic melt wrapping fibers, and this layer is characterized by a large area of the exposed carbon layer, so the Si element content is significantly lower than that of the ablation layer. In contrast, the Si element contents of the pyrolysis layer and the original layer are considerably lower than those of the ablation layer and the carbonized layer. The detected Si element mainly comes from the Si-modified phenolic resin body and some exposed fibers, so the content is at a low level.

**Figure 10:**
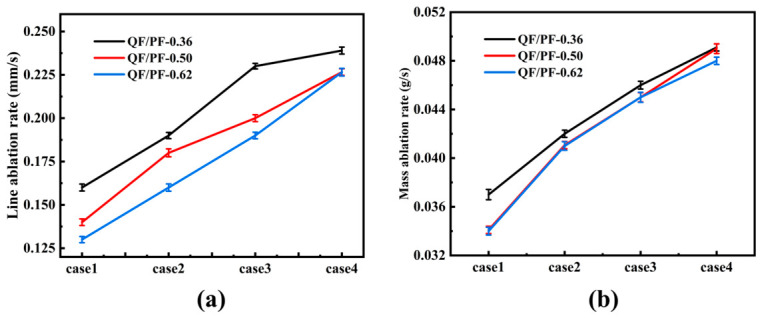
Ablation rates of phenolic aerogel composites with three fiber densities under case 1–case 4. (**a**) Linear ablation rate. (**b**) Mass ablation rate.

By adjusting the oxygen and acetylene flow rates to simulate four ablation environments with different intensities, the ablation resistance of the composites with three fiber densities is systematically evaluated, and the results are displayed in Figure 10. The linear ablation rate and mass ablation rate of the three composites increase with the increase in oxygen and acetylene flow rates. Among them, the optimal linear ablation performance is exhibited by QF/PF-0.62 under all four ablation conditions, followed by QF/PF-0.50, while the poorest linear ablation performance is exhibited by QF/PF-0.36. It is worth noting that the linear ablation rate of QF/PF-0.62 shows an approximately linear growth trend under the four ablation environments. QF/PF-0.50 reveals a Z-shaped change characteristic and rises rapidly during the environmental transition from case 1 to case 2 and from case 3 to case 4. The linear ablation rate of QF/PF-0.36 rises rapidly in the stage from case 2 to case 3, and the growth rate slows down significantly in the stage from case 3 to case 4.

In contrast, the growth curves of the mass ablation rate of the three composites under the four ablation environments are approximately parabolic, and the mass ablation rates among the materials are similar overall, with small numerical differences. The small difference in the mass ablation rate is due to gasification, sublimation and thermal decomposition of the resin matrix at high temperatures, which are the main reasons for mass loss during ablation. The three composites use exactly the same raw materials, so the mass ablation rate difference is small. The linear ablation rate of the three composites is quite different, which indicates that fiber density has an important influence on the linear ablation rate of composites. The linear ablation rates of the three composites under four ablation conditions are depicted in Figure 10. Under case 1, case 2, case 3 and case 4 conditions, the linear ablation rates are measured to be 0.16, 0.19, 0.23 and 0.239 mm/s for QF/PF-0.36; 0.14, 0.18, 0.20 and 0.226 mm/s for QF/PF-0.50; and 0.13, 0.16, 0.19 and 0.226 mm/s for QF/PF-0.62, respectively. Compared with QF/PF-0.36, the linear ablation rate is reduced by 12.5%, 5.3%, 13.0% and 5.4% for QF/PF-0.50, and by 18.8%, 15.8%, 17.4% and 5.4% for QF/PF-0.62 under the four working conditions, respectively. The higher the fiber density, the more layers of fiber fabric and mesh tires in the fiber preform, and the more fibers in the composite. During ablation, more fiber fabric and mesh tires absorb heat through phase change, and their molten state can form a sufficient liquid-phase barrier on the ablation surface, effectively blocking the invasion of high-temperature gas into the material, thereby slowing down the linear ablation process. Therefore, with the increase in fiber density, the overall heat absorption capacity of the material is markedly enhanced, and more ceramic coating layers can be generated, further improving the resistance to high-speed air flow erosion and finally leading to a significant reduction in the linear ablation rate.

## 3. Conclusions

In this paper, quartz fiber-reinforced Si-modified phenolic aerogel composites with different fiber densities are fabricated. The composites achieve an effective integration of high-efficiency thermal insulation, excellent load-bearing capacity and superior ablation resistance, providing promising candidates with balanced performance for high-temperature thermal protection applications. QF/PF forms a highly uniform nanoporous structure with an average pore size stable in the range of 73–76 nm and a material density of 0.71–0.85 g/cm^3^. Benefiting from the efficient inhibition of heat transfer by the nanoporous structure, QF/PF composites demonstrate a thermal conductivity lower than 0.122 W/(m·K) in the whole temperature range of 150–300 °C, and the thermal conductivity decreases with the increase in fiber density. Among them, the best thermal insulation performance in the whole temperature range is presented by QF/PF-0.62 with high fiber density, which has a thermal conductivity of only 0.086 W/(m·K) at 150 °C and as low as 0.103 W/(m·K) at 300 °C, showing excellent wide-temperature thermal insulation performance. The outstanding thermal insulation performance originates from the efficient inhibition of heat transfer by the nanoporous structure inside the composites, and the increase in fiber density further optimizes the heat transfer resistance of the material.

According to thermal stability test data, a residual carbon rate of more than 70% is maintained by QF/PF composites in the temperature range of 0–800 °C, and favorable high-temperature thermal stability is achieved. This excellent thermal stability is attributed to the fact that quartz fibers remain almost unchanged within the test temperature range, which effectively restrains the mass loss caused by thermal decomposition and quality reduction in the resin matrix. The thermal stability is improved with the increase in fiber density.

In terms of mechanical properties, good tensile properties are demonstrated by the composite, which are positively correlated with fiber density. Taking QF/PF-0.62 with high fiber density and QF/PF-0.36 with low fiber density as examples, at 20 °C, the tensile strength of QF/PF-0.62 reaches 129 MPa, which is 79.4% higher than that of QF/PF-0.36. In the 300 °C high-temperature environment, QF/PF-0.62 still maintains a tensile strength of 102 MPa, and the strength improvement range compared with QF/PF-0.36 further expands to 122.2%. Although the tensile strength of all QF/PF composites declines to a certain extent with the temperature rising from 20 °C to 300 °C, the attenuation range decreases significantly with the rise in fiber density. The enhanced tensile properties and weakened high-temperature attenuation are ascribed to the improved structural integrity and stress transfer efficiency of the composites induced by high fiber density, which effectively alleviates the adverse effects of internal gas accumulation and defect expansion caused by high temperature on the load-bearing performance of the materials.

According to the ablation resistance test results, the ablation resistance of QF/PF composites can be significantly optimized by increasing the preform fiber density, as reflected by the obvious reduction in the linear ablation rate. Under the ablation condition with an oxygen flow rate of 950 L/h and an acetylene flow rate of 700 L/h, the linear ablation rate of QF/PF-0.62 is only 0.13 mm/s, which is 23.1% lower than that of QF/PF-0.36 (0.16 mm/s). Even in the harsh ablation environment with an oxygen flow rate of 1350 L/h and an acetylene flow rate of 1000 L/h, its linear ablation rate is only 0.225 mm/s, which is 6% lower than that of QF/PF-0.36 (0.24 mm/s). During ablation, the ceramic melt generated on the material surface can markedly inhibit the pyrolysis rate of the resin matrix and enhance the resistance of the material to high-temperature gas scouring, thereby improving the overall ablation resistance of the composite. In summary, QF/PF composites have excellent wide-temperature thermal insulation performance, stable high-temperature bearing performance and good ablation resistance, highlighting their broad application prospects in the aerospace field.

## 4. Materials and Methods

### 4.1. Raw Materials

Composites were prepared using quartz fiber knitted felts as reinforcements and nanoporous Si-modified phenolic resin as the matrix. The phenolic resin was synthesized from phenol, formaldehyde and methyltriethoxysilane with ethanol as the solvent, which was purchased from Wuhan Yingcheng Lifa Chemical Co., Ltd., Wuhan, China. The key performance parameters of the resin are as follows: density 0.97 g/cm^3^, viscosity 19 mPa·s, and solid content 36%. Hexamethylenetetramine from the same manufacturer was used as the curing agent [44]. The reinforcement phase adopted quartz fiber knitted felts provided by Wuhan Huanyu Feilong New Material Technology Co., Ltd., Wuhan, China, which were prepared by alternately stacking fiber fabric and mesh tires. To study the influence of fiber density on the properties of composites, three kinds of knitted felts with densities of 0.36 g/cm^3^, 0.50 g/cm^3^ and 0.62 g/cm^3^ were selected.

### 4.2. Preparation of Composites

Phenolic aerogel composites were prepared by the RTM process. The specific preparation process was as follows: First, quartz fiber knitted felt preforms with a size of 300 × 300 × 5 mm were placed in the mold cavity, and the mold was closed after checking that the surface of the knitted felt was flat and wrinkle-free. Then, the phenolic resin adhesive system was prepared. Phenolic resin adhesive and hexamethylenetetramine curing agent were weighed at a weight ratio of 4:1 and stirred by a stirrer (HJ-2A8, Changzhou Liangyou Instrument Co., Ltd., Changzhou, China) for 5 min until completely homogeneous. At room temperature, the prepared adhesive was injected into the closed mold by high-pressure injection (HHR TM-06Z, Beijing Hongrun Zhunze Technology Development Co., Ltd., Beijing, China) at 0.4 MPa to ensure that the resin fully infiltrated the fibers. The curing and drying stages were carried out in a programmed temperature control oven (TD-1AG, Ningbo Dongfang Jiaxun Heating Equipment Co., Ltd., Ningbo, China). The curing process lasted 20 h at 100 °C, and the drying process lasted 24 h at 60 °C to effectively remove reaction by-products and residual solvents. According to the difference in quartz fiber preform density, the obtained composites were named as follows. The composite with a density of 0.36 g/cm^3^ is recorded as QF/PF-0.36. The composite with a density of 0.50 g/cm^3^ is referred to as QF/PF-0.50. The composite with a density of 0.62 g/cm^3^ is denoted as QF/PF-0.62.

### 4.3. Characterization

Tensile property measurement: Tensile property measurements of composites were carried out on a CMT6103 electronic universal testing machine (Meters Industrial Systems, Northbrook, IL, USA). Tensile properties at four temperatures of 20 °C, 100 °C, 200 °C and 300 °C were determined, and the load loading rate was 5 mm/min. Five parallel specimens were tested for each condition.

Thermal conductivity characterization: A Hot Disk TPS 2500S thermal conductivity analyzer (Hot Disk, Gothenburg, Sweden) was used to characterize the thermal conductivity at four temperatures of 150 °C, 200 °C, 250 °C and 300 °C. Three parallel specimens were tested for each temperature condition.

Specific heat capacity measurement: An STA 449F3 simultaneous thermal analyzer (NETZSCH, Selb, Germany) was used to measure the specific heat capacity, and the test temperature range was 100–800 °C. Three parallel specimens were adopted for each measurement.

Thermogravimetric test: A Rigaku comprehensive thermal analyzer (Rigaku, Shibuya-ku, Tokyo, Japan) was used for thermogravimetric tests at 0–800 °C, with a test heat flow rate of 50 mL/min and a heating rate of 10 °C/min. Three parallel specimens were tested for each measurement.

Microstructure analysis: Microstructure analysis was performed to characterize the pore distribution by an AutoPore IV 9510 mercury porosimeter (Micromeritics Instrument Co., Norcross, GA, USA), measure the sample density by an AccuPyc 1340 instrument (Micromeritics Instrument Co., Norcross, GA, USA), observe the morphology by a Sigma 300 scanning electron microscope (Zeiss, Oberkochen, Germany), and analyze the elemental composition by an Ultima IV X-ray diffractometer (Rigaku, Shibuya-ku, Tokyo, Japan), with a scanning speed of 2°/min and a scanning range of 10–80°.

Ablation resistance evaluation: Ablation resistance was evaluated by an HDQCS-N oxyacetylene ablation system (Xi’an HanDa Measurement And Control Technology Co., Ltd., Xi’an, China) to simulate different thermal environments. Three parallel specimens were used for each measurement.

## Figures and Tables

**Table 1:** Mass loss and characteristic temperatures of phenolic aerogel composites with three fiber densities.

Typology	T_5%_ (°C)	T_10%_ (°C)	T_d·max_ (°C)	R_800°C_ (%)
QF/PF-0.36	169.3 ± 1.11 ^a^	300.8 ± 1.65 ^a^	561.3 ± 0.68 ^a^	71.7 ± 0.49 ^a^
QF/PF-0.50	179.5 ± 0.91 ^b^	370.1 ± 1.24 ^b^	560.3 ± 0.72 ^a^	75.68 ± 0.58 ^b^
QF/PF-0.62	343.8 ± 1.37 ^c^	498.1 ± 1.32 ^c^	545.9 ± 1.12 ^b^	79.62 ± 0.63 ^c^

Note: Data are mean ± SD (n = 3). Different letters in the same column indicate a significant difference at *p* < 0.05.

**Table 2:** Thermal conductivity of phenolic aerogel composites with three fiber densities at different temperatures.

Typology	150 °C (W/m·K)	200 °C (W/m·K)	250 °C (W/m·K)	300 °C (W/m·K)
QF/PF-0.36	0.094 ± 0.0011 ^a^	0.098 ± 0.0013 ^a^	0.112 ± 0.0015 ^a^	0.121 ± 0.0012 ^a^
QF/PF-0.50	0.091 ± 0.0012 ^b^	0.095 ± 0.001 ^b^	0.108 ± 0.0017 ^b^	0.116 ± 0.0022 ^b^
QF/PF-0.62	0.086 ± 0.0013 ^c^	0.089 ± 0.0023 ^c^	0.098 ± 0.0017 ^c^	0.103 ± 0.0021 ^c^

Note: Data are mean ± SD (n = 3). Different letters in the same column indicate a significant difference at *p* < 0.05.

**Table 3:** Improvement in thermal conductivity of QF/PF-0.36 compared with QF/PF-0.62 at different temperatures.

150 °C (W/m·K)	200 °C (W/m·K)	250 °C (W/m·K)	300 °C (W/m·K)
0.008 ± 0.0017 ^a^	0.009 ± 0.0026 ^b^	0.014 ± 0.0023 ^c^	0.018 ± 0.0024 ^d^

Note: Data are mean ± SD (n = 3). Different letters in the same row indicate a significant difference at *p* < 0.05.

**Table 4:** Tensile strength of phenolic aerogel composites with three fiber densities at different temperatures.

Tensile Strength	20 °C (MPa)	100 °C (MPa)	200 °C (MPa)	300 °C (MPa)
QF/PF-0.36	71.9 ± 1.33 ^a^	58.9 ± 1.25 ^a^	50.8 ± 1.25 ^a^	45.9 ± 0.99 ^a^
QF/PF-0.50	110 ± 1.59 ^b^	104 ± 1.61 ^b^	94.1 ± 1.48 ^b^	84.4 ± 1.44 ^b^
QF/PF-0.62	129 ± 1.58 ^c^	122 ± 1.46 ^c^	110 ± 1.43 ^c^	102 ± 1.57 ^c^

Note: Data are mean ± SD (n = 5). Different letters in the same column indicate a significant difference at *p* < 0.05.

**Table 5:** Improvement in tensile strength of QF/PF-0.62 compared with QF/PF-0.36 at different temperatures.

20 °C (%)	100 °C (%)	200 °C (%)	300 °C (%)
79.4 ± 3.23 ^a^	107.1 ± 3.98 ^b^	116.5 ± 3.87 ^c^	122.2 ± 3.21 ^d^

Note: Data are mean ± SD (n = 5). Different letters in the same row indicate a significant difference at *p* < 0.05.

**Table 6:** Attenuation of tensile strength of three composites from 20 °C to 300 °C.

QF/PF-0.36 (%)	QF/PF-0.50 (%)	QF/PF-0.62 (%)
36.16 ± 2.4 ^a^	23.27 ± 1.98 ^b^	20.93 ± 1.75 ^c^

Note: Data are mean ± SD (n = 5). Different letters in the same row indicate a significant difference at *p* < 0.05.

**Table 7:** Test items of ablation rate.

Condition	Oxygen Flow Rate (L/h)	Acetylene Flow Rate (L/h)
case 1	950	700
case 2	1080	800
case 3	1200	900
case 4	1350	1000

**Table 8:** Atomic percentages of SiO_2_, SiC and C in the carbonized layer on the ablation surfaces of three composites.

Atomic Percentage	C_SiO_2__ (%)	C_SiC_ (%)	C_C1_ (%)
QF/PF-0.36	15.14	0.6	53.38
QF/PF-0.50	14.915	4.125	47.005
QF/PF-0.62	24.42	2.58	21.58

## Data Availability

The data presented in this study are available on request from the corresponding author.
